# Abnormal cerebellar-prefrontal cortical pathways in obstructive sleep apnea with/without mild cognitive impairment

**DOI:** 10.3389/fnins.2022.1002184

**Published:** 2022-10-20

**Authors:** Yongqiang Shu, Liting Chen, Kunyao Li, Haijun Li, Linghong Kong, Xiang Liu, Panmei Li, Wei Xie, Yaping Zeng, Dechang Peng

**Affiliations:** ^1^Department of Radiology, The First Affiliated Hospital of Nanchang University, Nanchang, Jiangxi, China; ^2^Medical Imaging Center, First Affiliated Hospital of Jinan University, Guangzhou, China

**Keywords:** obstructive sleep apnea, mild cognitive impairment, fMRI, dynamic, amplitude of low-frequency fluctuation

## Abstract

Obstructive sleep apnea (OSA), a common respiratory sleep disorder, is often associated with mild cognitive impairment (MCI), which is a precursor stage to Alzheimer’s disease (AD). However, the neuroimaging changes in patients with OSA with/without MCI are still under discussion. This study aimed to investigate the temporal variability of spontaneous brain activity in OSA. Fifty-two OSA patients (26 with OSA with MCI (OSA-MCI), 26 OSA without MCI (OSA-nMCI), and 26 healthy controls (HCs) underwent MRI scans and scale questionnaires. A dynamic amplitude of low-frequency fluctuation (dALFF) evaluation was performed to examine the time-varying nature of OSA-MCI and OSA-nMCI. Compared with OSA-MCI, OSA-nMCI had increased dALFF in the posterior cerebellar and right superior frontal gyrus; compared with HCs, OSA-nMCI patients showed increased dALFF in the right posterior cerebellum. A positive correlation between the bilateral posterior cerebellar lobes and right superior frontal gyrus was observed in OSA-MCI patients; however, in OSA-nMCI patients, a positive correlation was observed only between the bilateral posterior cerebellar lobes. The dALFF value of the left posterior cerebellar lobe was positively correlated with the apnea-hypopnea index (AHI), epworth sleepiness scale (ESS) score, and arousal index in OSA-nMCIs, while the dALFF value of the right posterior cerebellum was positively correlated with the AHI and negatively correlated with the lowest oxygen saturation (SaO2). This study argues that OSA-nMCIs and OSA-MCIs exhibit different temporal variabilities in dynamic brain functions, OSA-nMCIs may have variable intermediate states. We concluded that the functional abnormalities of the cerebellar-prefrontal cortex pathway in OSA-MCIs may cause cognitive impairment with OSA.

## Introduction

Obstructive sleep apnea (OSA) is a common sleep disorder characterized by partial or complete upper airway stenosis leading to decreased airflow, intermittent hypoxia, and sleep fragmentation (Jordan et al., 2014). Epidemiological studies have shown that 5.7–6.0% of middle-aged men and 2.4–4.0% of middle-aged women were affected by OSA (Franklin and Lindberg, 2015). OSA is not only highly prevalent, but also associated with chronic intermittent hypoxia (Kang et al., 2009; Ju et al., 2013), sleep fragmentation (Montplaisir et al., 1995; Pase et al., 2017), oxidative stress (Ng et al., 2010; Shiota et al., 2013), and cardiovascular complications (Jellinger, 2007; Xu et al., 2011), and have a higher risk for cognitive impairment that mainly manifests as memory loss, decreased executive ability, and inattention, which are major risk factors for Alzheimer disease’s (AD) (Bennett et al., 1999; Verstraeten, 2007).

Recently, the close relationship between OSA and AD has been noticed by epidemiology. Both often co-exist in elderly patients and mutually reinforce each other. OSA patients are at significantly higher risk for AD and patients with AD are about five times more likely to have obstructive sleep apnea than healthy individuals of similar age (Emamian et al., 2016). Mild cognitive impairment (MCI) is usually considered a prodromal stage of AD. MCI is a state of abnormal cognitive function, but basic normal life is not disturbed. Although MCI patients do not meet the diagnostic criteria for AD, most do not avoid the development of AD (Skiba et al., 2020; Li et al., 2021a). Currently, there is no specific treatment for MCI (Petersen et al., 2018), although MCI cannot be completely cured, the Collaboration for Alzheimer’s Prevention recommends that the progression of MCI to AD can be controlled by controlling several important factors such as hypoxia and sleep fragmentation. For example, a retrospective study showed that the age of onset of MCI/AD in OSA patients was younger than that in non-OSA patients, and that treatment recommended by the Collaboration for Alzheimer’s Prevention effectively delayed the onset of MCI by approximately 10 years (Osorio et al., 2015). Considering the importance of MCI in the development of AD and evolutionary trends in OSA, early identification and prevention of MCI for OSA patients are of great significance. At present, a large number of functional magnetic resonance imaging (MRI) studies have explored neuroimaging changes in OSA and MCI patients, but the neuropathological transition mechanism between OSA and MCI is still unclear. Therefore, it is urgent to explore the changes in the brain activity of OSA without MCI (OSA-Nmci) patients and OSA with MCI (OSA-MCI) patients.

As a non-invasive neuroimaging technique, functional MRI has been widely used for mental illnesses and sleep related disorders. Neuroimaging studies based on functional MRI have confirmed that OSA patients and MCI patients have extensive abnormal brain activity (Tao et al., 2019; Yu et al., 2019). Peng et al. (2014) found that OSA patients had abnormalities in the default mode network (DMN), including the medial frontal gyrus, angular gyrus, and precuneus. Simultaneously, intermittent hypoxia was negatively correlated with brain activity in the bilateral frontal and left parietal lobes. Therefore, intermittent hypoxia may be one of the main causes of the damage of network function. Liu Y. T. et al. (2018) found that the voxel-mirrored homotopic connectivity values of the bilateral talus gyrus and precuneus cortex were significantly increased and were positively correlated with the apnea-hypopnea index (AHI) in OSA patients, indicating that AHI changes may have a vital role in the development of OSA. A functional connectivity (FC) studies have shown that the FC of the medial temporal lobe subintervals was enhanced, whereas the FC between the medial temporal lobe and the DMN was reduced in MCI patients (Das et al., 2013). Another study about MCI exhibited decreased degree centrality within DMN, including the bilateral precuneus, angular gyrus, and medial frontal gyrus (Li et al., 2021). These studies have shown that patients with OSA and MCI have experienced extensive changes in brain function, and that the main abnormal brain areas are mostly concentrated in the default network areas, such as the medial prefrontal cortex, posterior cingulate gyrus, precuneus, and bilateral temporal cortex. These studies suggest that there is some similarity in brain activity changes between OSA and MCI patients. However, it is unfortunate that most studies have focused on functional abnormalities of OSA or MCI and did not effectively link the 2. Considering the close interdependence of the OSA and MCI, it is inappropriate to ignore the link between OSA and MCI.

Li et al. (2015) found that the amplitude of low-frequency fluctuation (ALFF) value decreased in the right precuneus and bilateral posterior cingulate gyrus, whereas the ALFF value of the left inferior frontal gyrus increased in OSA patients and they implied that the default network and prefrontal cortex have an important role in the changes of craniocerebral function in OSA patients. In the follow-up OSA frequency dependence study, Haijun et al. (2016) found that compared with the slow-5 frequency band, the ALFF value increased in the left superior temporal pole and decreased in the left orbital, inferior frontal gyrus, bilateral middle frontal gyrus in the slow-4 frequency band. The slow-4 band showed more extensive functional changes than the slow-5 band. However, ALFF which based on resting-state functional MRI was ignored dynamic changes in brain neural activity. In contrast to the resting state, the dynamic state can capture the various states that occur repeatedly in brain activity (Xie et al., 2018), supplement static defects, and have increased sensitivity compared to that during the resting state, with good stability (Liu et al., 2017; Liu J. et al., 2018), and reproducibility (Shen et al., 2017). Dynamic ALFF (dALFF) evaluations combine ALFF with the sliding time window method to explore the time variability of spontaneous brain activity. Therefore, dALFF evaluations have been widely used for AD (Li et al., 2021b), primary insomnia (Meng et al., 2020), and other diseases.

In this study, the dALFF was evaluated to explore the temporal variability of spontaneous brain activity among healthy controls (HCs), OSA-nMCI patients, and OSA-MCI patients. We hypothesized that OSA-MCI and OSA-nMCI would have different dALFF patterns compared to those of HCs. and the relationship between dALFF values of brain areas and the cognition and behavior were analyzed.

## Materials and methods

### Participants

Fifty-two OSA patients (26 OSA-MCI and 26 OSA-nMCI patients) and 26 HCs were recruited from the First Affiliated Hospital of Nanchang University, Jiangxi Province, China. The criteria for OSA were as follows: AHI ≥ 30; male; age 18–65 years; right hand dominance; with typical clinical symptoms and not match exclusion criteria; The criteria for HC were as follows: AHI < 5; male; age 18–65 years; right hand dominance; without typical clinical symptoms and not match exclusion criteria.

The exclusion criteria of OSA and HC were as follows: other sleep disorders, including primary insomnia and sleep deprivation; intracranial organic lesions, such as tumors, traumatic brain injury; chronic diseases such as hypertension and diabetes; nervous system diseases such as schizophrenia, depression.

All MCIs were accurately determined by two experienced physicians. The inclusion criteria were as follows: impaired memory performance according to a normalized objective verbal memory delayed recall test; recent history of symptomatic worsening of memory; normal or near-normal performance according to global cognitive tests, including a MoCA score from 18 to 26; normal or near-normal performance according to a scale of activities of daily living; and absence of dementia.

This study was conducted in accordance with the Declaration of Helsinki and approved by the Medical Ethics Committee of the First Affiliated Hospital of Nanchang University. Informed consent was obtained from all participants.

### Overnight polysomnography

Polysomnography (PSG) was performed the day before the MRI scan. All subjects were asked to avoid using sleeping pills, alcohol, and caffeine. A physiological monitoring system (Alice 5 LE; Respironics, Orlando, FL, USA) was used to check the subject’s breathing and sleep status at the Sleep Monitoring Center of the First Affiliated Hospital of Nanchang University. The evaluation time was from 10:00 p.m. to 6:00 a.m. the next morning. Oxygen saturation (SaO2), electrocardiography, standard electroencephalography, chin electromyography, electrooculography, thoracic and abdominal respiratory movements, oral and nasal airflow, snoring, and body position were recorded.

According to the guidelines of the American Academy of Sleep Medicine, obstructive apnea is defined as the continuous absence of airflow or a reduction in airflow ≥90% for at least 10 s associated with evident respiratory effort. Hypopnea was defined as a reduction in airflow of ≥30 with ≥4% oxygen desaturation or electroencephalographic arousal. The AHI was obtained from the mean duration of apnea and hypopnea each hour during sleep. OSA was diagnosed when the AHI was ≥5; severe OSA was diagnosed when the AHI was >30.

### Cognition and sleep assessment

Obstructive sleep apnea without MCI and OSA-MCI was distinguished using the Montreal Cognitive Assessment (MoCA, Chinese version)^1^ with eight cognitive items, including executive function, memory, attention, calculation, abstraction, naming, language, and orientation. OSA patients with MoCA scores from 18 to 26 were defined as OSA-MCI. The ESS was used to assess the sleep quality and daytime sleepiness.

### Imaging data acquisition

The MRI data were collected using a 3.0T MRI system with an eight-channel phased-array head coil at the First Affiliated Hospital of Nanchang University. During the MRI scanning, all subjects were placed in the supine position, with the head in a neutral position and placing foam pads to reducing head movement. Each participant was instructed to keep the eyes closed, to relax, to remain awake, and to not think about anything. Resting-state functional images with a gradient-recalled echo-planar imaging (EPI) sequence were acquired using the following parameters: repetition time (TR), 2000 ms; echo time, 40 ms; flip angle, 90^°^; slice thickness/gap, 4.0/1 mm; field of view, 240 mm × 240 mm; in-plane resolution, 64 × 64; 30 axial slices covering the whole brain; and 240 volumes acquired within 8 min. In addition, we acquired high-resolution brain structural images for each subject using a *T*1-weighted 3D MP-RAGE sequence (TR, 1,900 ms; echo time, 2.26 ms; flip angle, 9^°^; matrix, 256 × 256; field of view, 240 mm × 240 mm; thickness, 1.0 mm; and 176 sagittal slices).

### Data preprocessing

Data processing was performed using Statistical Parametric Mapping (SPM12)^2^ and Data Processing and Analysis Assistant for Resting-State Brain Imaging 6.0 (DPARSF6.0),^3^ which is based on the MATLAB toolbox. The first 10 time points were discarded for each subject after all the DICOM data were converted to NIFIT files. The remaining 230 volumes were corrected for the acquisition time. Head movement correction was performed to correct for small head movements during MRI scans. Any subject who moved more than 1.5 mm maximum displacement in any of the directions (*x*, *y*, *z*), or more than 1.5°of angular rotation in any axis was excluded. The individual high-resolution *T*1-weighted structural images were co-registered to the mean of the realigned EPI images and segmented into gray matter, white matter, and cerebrospinal fluid. The functional images after correction for head movement were registered to the Montreal Neurological Institute space by the DARTEL tool and then re-sampled with a resolution of 3 mm × 3 mm × 3 mm. The resulting images were spatially smoothed with the full width at half maximum of 6 mm. To reduce the effects of confounding factors, the nuisance covariate effects of six parameters of head were evaluated.

### Dynamic amplitude of low-frequency fluctuations analysis

Temporal dynamic analysis toolkits based on DPABI were used to analyze the dALFF. When the sliding time window method is used, the window width and step size are important influences to explore the temporal variability of brain functional activity. Large widths and length are likely to cause the dynamics to be ignored; however, if they are too small, then strong variability cannot be discovered. Recent research has shown that the minimum window length should be larger than 1 fmin, where fmin is the minimum frequency of the time series. Therefore, a sliding window length with a TR of 30 and a step length with a TR of 1 were used in this study. The 230 times points reserved for each subject were divided into 201 sliding windows, and the low-frequency amplitude of each sliding window was calculated. To improve the normality of the correlation distribution, Z-standardization was applied to all maps. To obtain the time dynamic changes in the ALFF diagrams under the sliding windows, we chose the coefficient of variation of low-frequency amplitude to represent the dALFF, which represents the temporal variability of the energy of the brain’s neural activity.

### Statistical analysis

Demographic and clinical variables were checked for normal distribution and homogeneity of variance in OSA-nMCI, OSA-MCI, and HC groups. Demographic and clinical variables were compared using analysis of variance (ANOVA) or Kruskal–Wallis test with IBM Statistical Package for the Social Sciences software (SPSS version 26.0; SPSS Inc., Chicago, IL, USA). The significance level was set at *p* < 0.05.

All whole-brain dALFF data were analyzed by an ANOVA to detect differences among the OSA-nMCI, OSA-MCI, and HC groups. Then, the abnormal brain regions among the groups were calculated using a *post hoc* analysis based on a two-sample *t*-test. Age, education level, and head motion were entered as nuisance covariates. The threshold for significance was set at *p* < 0.05. Multiple comparisons were corrected using the Gaussian random field method (voxel level, *p* < 0.005; cluster level, *p* < 0.05).

Furthermore, a Pearson correlation analysis was performed for the OSA-nMCI and OSA-MCI patients to assess the correlations between the clinical variables and dALFF values of the abnormal brain areas. The significance level was set at *p* < 0.05.

## Results

### Demographic and clinical data

Demographic and clinical data and *Post hoc* comparisons from the OSA-nMCI, OSA-MCI, and HC groups are summarized in Table 1. As expected, compared with HCs, OSA-nMCIs, and OSA-MCIs showed significantly higher body mass index, AHI, Total sleep time, *N*1 stage, SaO2 < 90%, arousal index, ESS scores and lower minimum SaO2, mean SaO2, Sleep efficiency, *N*3 stage, rapid eye movement (*p* < 0.001). There were no significant differences in age (ANOVA test, *p* = 0.436) or education level (*p* = 0.452).

**TABLE 1:** Demographic and clinical information of the subjects.

Characteristic	OSA-nMCIs (*N* = 26)	OSA-MCIs (*N* = 26)	HCs (*N* = 26)	F/H	*p*-Value
Age (year)	36.15 ± 9.48	39.26 ± 10.25	39.84 ± 11.18	1.66^F^	0.961
BMI (Kg/m^2^)	27.12 ± 4.00	27.27 ± 2.77	19.70 ± 0.98	51.48^H^	0.000^ab^
AHI (/h)	54.05 ± 19.06	56.79 ± 24.73	2.35 ± 1.21	51.35^H^	0.000^ab^
Education (year)	12.92 ± 3.17	11.61 ± 2.80	12.23 ± 3.76	1.59^H^	0.452
LSaO2 (%)	65.00 ± 11.73	70.65 ± 12.87	94.03 ± 3.25	52.91^H^	0.000^bc^
MSaO2 (%)	90.54 ± 4.91	92.00 ± 3.88	97.11 ± 2.00	34.68^H^	0.000^bc^
Total sleep time (min)	450.6 ± 64.38	436.1 ± 44.89	401.8 ± 12.40	17.40^H^	0.000^ab^
Sleep efficiency (%)	0.864 ± 0.144	0.849 ± 0.151	91.13 ± 3.201	51.34^H^	0.000^bc^
N1 stage (%)	30.51 ± 17.78	28.57 ± 16.69	10.48 ± 3.59	29.15^H^	0.000^ab^
N2 stage (%)	39.50 ± 15.41	40.00 ± 12.61	38.99 ± 6.435	0.04^H^	0.98
N3 stage (%)	22.05 ± 18.37	22.62 ± 17.40	30.63 ± 5.38	11.40^H^	0.003^ab^
REM (%)	7.74 ± 7.31	8.10 ± 8.93	20.46 ± 6.36	30.46^H^	0.000^ab^
SaO2 < 90%	25.60 ± 18.35	25.39 ± 20.75	0.23 ± 0.17	51.01^H^	0.000^ab^
AI (/h)	38.20 ± 21.91	36.80 ± 23.80	11.65 ± 2.97	34.26^H^	0.000^ab^
MOCA	27.34 ± 1.26	22.84 ± 2.80	27.57 ± 1.67	49.99^H^	0.000^bc^
ESS	11.61 ± 4.15	11.96 ± 4.13	3.03 ± 1.82	46.30^H^	0.000^ab^
Head movement	0.20 ± 0.10	0.16 ± 0.07	0.11 ± 0.07	8.481^F^	0.000^ab^

BMI, body mass index, AHI, apnea hypopnea index; LSaO2, minimum blood oxygen saturation; MSaO2, average blood oxygen saturation; REM, rapid eye movement; SaO2 < 90%, percentage of total sleep time with oxygen saturation less than 90; AI, arousal index; MOCA, Montreal Cognitive Assessment; ESS, Epworth Sleepiness Scale; F, analysis of variance; H, Kruskal–Wallis test.

^*a*^Significant difference between HCs and OSA-nMCIs in *post hoc* analysis.

^*b*^Significant difference between HCs and OSA-MCIs in *post hoc* analysis.

^*c*^Significant difference between OSA-nMCIs and OSA-MCIs in *post hoc* analysis.

### Dynamic amplitude of low-frequency fluctuation variability

**FIGURE 1:**
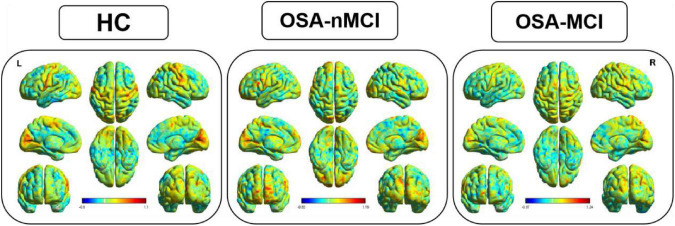
The distribution patterns of dynamic amplitude of low-frequency fluctuation (dALFF) in the healthy control (HC), OSA without MCI (OSA-Nmci), and OSA with MCI (OSA-MCI) groups.

**FIGURE 2:**
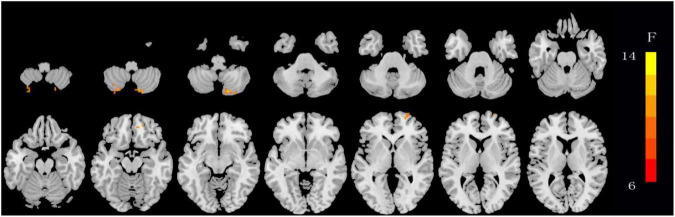
Maps showing differences in dynamic amplitude of low-frequency fluctuation (dALFF) in OSA without MCI (OSA-nMCI), OSA with MCI (OSA-MCI), and healthy control (HC) groups. The hot color indicates significantly differences dALFF brain area. Differences between the groups were calculated using analysis of variance (ANOVA) with the threshold set at voxel *P* < 0.05 and cluster *P* < 0.005 with GRF correction.

**FIGURE 3:**
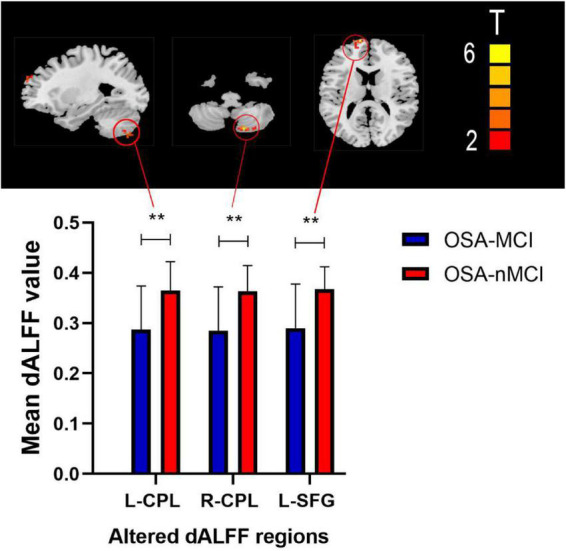
Maps showing differences in dynamic amplitude of low-frequency fluctuation (dALFF) in the OSA without MCI (OSA-nMCI) with OSA with MCI (OSA-MCI) groups. Differences between the groups were calculated using *post hoc* analysis based on a two-sample *T*-test with the threshold set at voxel *P* < 0.05 and cluster *P* < 0.005 with GRF correction. ** means that there are significant difference between the groups.

**FIGURE 4:**
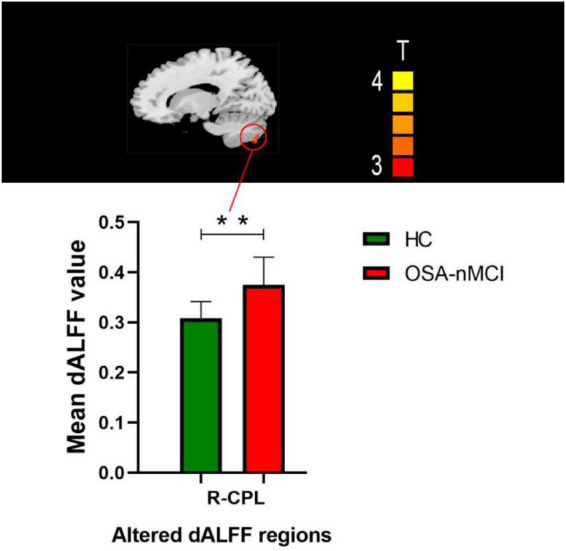
Maps showing differences in dynamic amplitude of low-frequency fluctuation (dALFF) in the OSA without MCI (OSA-nMCI) with healthy control (HC) groups. Differences between the groups were calculated using *post hoc* analysis based on a two-sample *T*-test with the threshold set at voxel *P* < 0.05 and cluster *P* < 0.005 with GRF correction. ** means that there are significant difference between the groups.

The ANOVA results revealed that the OSA-nMCIs, OSA-MCIs, and HCs groups exhibited significant dALFF variability in the bilateral cerebellum posterior lobe, right medial frontal gyrus, and bilateral superior frontal gyrus (Figure 1 and Table 2). For the voxel-based zCVALFF maps, one-sample *t*-tests were to identify the spatial distribution in three groups, respectively (Figure 2). Compared with OSA-nMCI patients, OSA-MCI patients exhibit decreased dALFF variability in the bilateral cerebellum posterior lobe and left superior frontal gyrus (Figure 3 and Table 2). Compared with HCs, OSA-nMCI patients had increased dALFF variability in the right cerebellar posterior lobe (Figure 4 and Table 2). However, there were no statistically significant differences in dALFF variability between the OSA-MCI and HC groups.

**TABLE 2:** Significant differences in dynamic amplitude of low-frequency fluctuation (dALFF) between patients with OSA without MCI (OSA-nMCI), OSA with MCIs (OSA-MCIs), and healthy controls (HCs).

	Brain area	Voxel	MNI			*F/T value*
			X	Y	Z	
ANOVA	L cerebellum posterior lobe	17	−30	−72	−54	8.408
	R cerebellum posterior lobe	27	15	−78	−45	14.000
	R medial frontal gyrus	10	9	45	−18	8.065
	L superior frontal gyrus	12	−12	69	15	11.583
	R superior frontal gyrus	12	24	66	3	9.765
OSA-nMCI vs. OSA-MCI	L cerebellum posterior lobe	29	−27	−72	−57	3.705
	R cerebellum posterior lobe	44	15	−78	−45	4.614
	R superior frontal gyrus	49	−12	69	15	4.327
OSA-nMCI vs. HC	R cerebellum posterior lobe	32	12	−75	−51	3.989

### Correlation analysis

**FIGURE 5:**
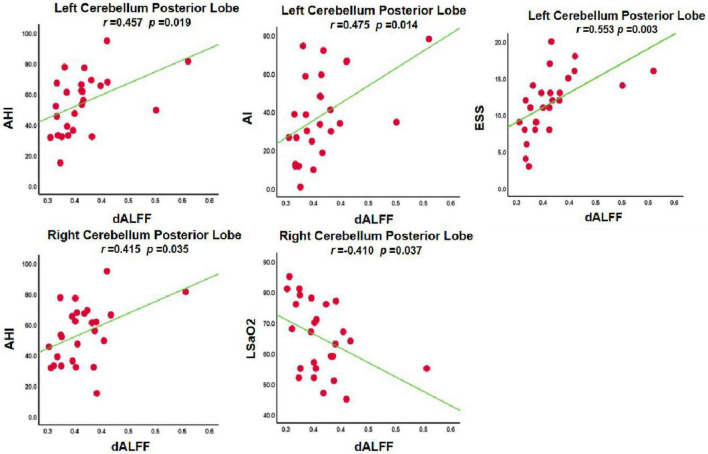
Correlations between dynamic amplitude of low-frequency fluctuation (dALFF) value and clinical information in OSA without MCI (OSA-nMCI).

**FIGURE 6:**
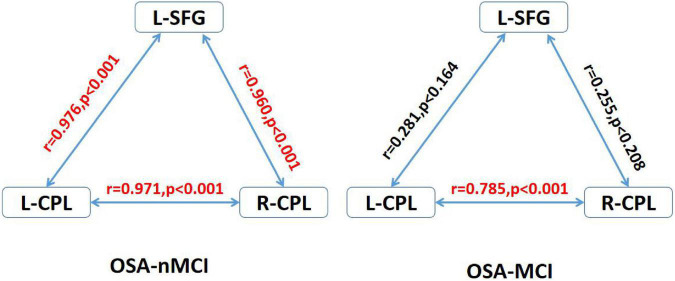
Correlations between differences regions in the OSA without MCI (OSA-nMCI) with OSA with MCI (OSA-MCI) groups. R-CPL, right cerebellum posterior lobe; L-CPL, left cerebellum posterior lobe; L-SFG, right superior frontal gyrus; red, significantly correlated.

In the OSA-nMCI group, the dALFF value of the left posterior cerebellum was significantly positively correlated with the AHI score (*r* = 0.457, *p* = 0.019), ESS score (*r* = 0.553, *p* = 0.003), and arousal index (*r* = 0.475, *p* = 0.014), whereas the dALFF value of the right posterior cerebellar lobe was positively correlated with the AHI score (*r* = 0.415, *p* = 0.035) and negatively correlated with the lowest SaO2 (*r* = −0.410, *p* = 0.037; Figure 5). Considering the existence of 1–2 outliers, we carefully checked the data and put it forward and re-performed the correlation analysis. The results show that they are roughly similar to the previous ones (detailed results are shown in Supplementary material 1), which also reflects the stability of our results. A correlation analysis of abnormal brain regions showed a strong correlation between the bilateral posterior cerebellum and right superior frontal gyrus (*r* = 0.971, *p* < 0.001; *r* = 0.943, *p* < 0.001, and *r* = 0.943, *p* < 0.001) in the OSA-MCI group, while a positive correlation between the bilateral posterior cerebellar lobes (*r* = 0.785, *p* < 0.001) was found in the OSA-nMCI group (Figure 6).

## Discussion

In the current study, the dALFF was used to evaluate the time variability of brain activity in OSA-nMCIs and OSA-MCIs. We found that there were significant differences in dALFF variability of the bilateral cerebellum posterior lobe, right medial frontal gyrus, and bilateral superior frontal gyrus among OSA-nMCI and OSA-MCI, and HCs. OSA-MCIs exhibited decreased dALFF variability in the bilateral cerebellum posterior lobe and left superior frontal gyrus. OSA-nMCIs showed increased dALFF variability in the right cerebellum posterior lobe, which indicated that changes in the temporal variability of brain function activity in these specific brain regions may lead to cognitive impairmen in OSA patients and may deepen our understanding of the mechanisms of cognitive impairment in OSA patients. The present study applies the dALFF approach to provide evidence that the progression of OSA-nMCI toward OSA-MCI may be associated with altered cerebellar-prefrontal cortical circuits, possibly caused by time-varying changes in its brain activity.

In the current study, compared with OSA-MCI patients and HCs, OSA-nMCI patients showed increased dALFF values in the bilateral posterior cerebellar lobe. Previous neuroimaging studies have shown that the cerebellum is generally attributed to motor regulation, besides, it is also closely connected to the limbic system and has a vital role in cognitive regulation (Liang and Carlson, 2020). There are extensive connections between the cerebellum and the cerebral cortex, and it is through this connection network that the effects of regulating cognitive function are achieved (Buckner, 2013; Stoodley and Schmahmann, 2018). The posterior lobe of the cerebellum, especially lobule VII and parts of lobule VI, is called the cognitive cerebellum and has an important role in the regulation of cognitive functions (Stoodley and Schmahmann, 2010). Li et al. (2016) found that the increased degree centrality of the bilateral posterior cerebellar lobe in OSA patients. Furthermore, Peng et al. (2014) found that the regional homogeneity value of the right posterior cerebellum in patients with OSA increased, which may be related to the regulation of breathing patterns The dALFF reflects the temporal variability of low-frequency amplitude; the higher the value, the greater the temporal variability of brain activity and the lower the stability. The increased dALFF in the bilateral posterior cerebellar lobe of OSA-nMCI patients in our study may imply that the cognitive function is in an intermediate or unstable state. From the perspective of the disease conversion status, the OSA-nMCI status is between that of OSA-MCI patients and HCs, proper treatment and ignoring the development of the disease may lead to two completely different outcomes. We also found that there was no significant difference between OSA-MCI patients and HCs, which may indicate that both are in a similarly stable state, thereby further supporting the speculation that OSA-nMCI patients are in an active intermediate state.

Our results show that of OSA-nMCI patients showed increased dALFF value in the superior frontal gyrus compared to OSA-MCI patients. The superior frontal gyrus is located in the prefrontal lobe which is an important part of the medial prefrontal cortex. According to Brodmann, the superior frontal gyrus spans four areas: BA6, BA8, BA9, and BA32 (Petrides and Pandya, 2002). Currently, its main functions are relatively clear, including work and memory, motor function, self-reflection, and increased-order cognitive processing (Briggs et al., 2020). The functional connection state of the forehead network reached its best cognitive state after rest. A high-density resting-state electroencephalography study confirmed that sleep deprivation has the strongest effect on the FC of the prefrontal cortex, which may also be the brain area most susceptible to sleep deprivation (Verweij et al., 2014). Insufficient sleep may lead to emotional disorders and abnormal cognitive function (Chen et al., 2017). Previous studies about the function (Zhou et al., 2020), metabolism (Yaouhi et al., 2009), and structure (Yaouhi et al., 2009) have confirmed that OSA patients have changes in the prefrontal lobe, suggesting that sleep fragmentation in OSA patients may lead to cognitive impairment. At the same time, the superior temporal gyrus belongs to both the DMN and the cognitive control network, which is the contact point of these two networks (Briggs et al., 2020). The DMN has an important role in a variety of complex cognitive behaviors, including memory and abstract thinking (Smallwood et al., 2021). Our previous studies have found that OSA patients showed disrupted FC and topological reorganization of the DMN and abnormal DMN FC may contribute to the topological configuration of the DMN and cognitive impairment in patients with OSA (Chen et al., 2018). The cognitive control network achieves its function through the regulation of cognitive function and emotion in other regions (Niendam et al., 2012). Studies have shown that the targeted application of transcranial magnetic stimulation to the cognitive control network, especially the dorsolateral prefrontal cortex, can improve the symptoms of mental illnesses, such as obsessive-compulsive disorder and post-traumatic stress disorder (Amidfar et al., 2019). This suggests that the abnormal changes in the superior frontal gyrus may be closely related to the deterioration of cognitive function in OSA-nMCI patients and OSA-MCI patients, and we assume that targeted non-invasive techniques such as transcranial magnetic stimulation may comprise possible treatment in the future.

In addition, we detected the correlation between the bilateral posterior cerebellar lobes and the left superior temporal gyrus among OSA-nMCI with OSA-MCI, and we found a positive correlation between the bilateral posterior cerebellar lobes in OSA-nMCI patients. The posterior cerebellar lobe and superior frontal gyrus are closely related and participate in the regulation of cognitive function. A study involving autistic mice confirmed the existence of the cerebellar-prefrontal cortex circuit, which regulates autism-related behaviors such as social and repetitive behaviors (Krienen and Buckner, 2009). FC analysis showed that cerebellar-prefrontal cortical circuit are also present in non-human primates (Allen et al., 2005). Changes in cerebellar function have been linked to involvement of the prefrontal cortex in cognitive disorders such as schizophrenia (Brady et al., 2019) and essential tremor (Passamonti et al., 2011). In summary, the strong correlation between the bilateral posterior cerebellar lobe and the left superior frontal gyrus in OSA-MCI patients may represent damage to the cerebellar-prefrontal cortex pathway in MCI patients, thus providing a new perspective for the study of cognitive impairment in OSA. In addition, there were statistically significant positive correlations among the left cerebellar hemisphere and AHI, ESS score, and microarousal index of OSA patients. The dALFF value in the right posterior cerebellar lobe was positively correlated with the AHI and the lowest SO2 score, which also suggested that the time variability of the posterior cerebellar lobe was significantly related to the clinical status of OSA patients, which may be a new imaging marker of OSA.

## Limitations

Our study had several limitations. First, we did not collect blood biochemical markers, such as Aβ42, T-tau, and P-tau. These indicators are risk factors for AD, and we are collecting them for follow-up study. Second, longitudinal research is extremely important to the study of the relationship between normal cognition and cognitive impairment; therefore, we will focus on longitudinal research in the future. Third, female patients were not included in this study, more female participants should be recruited in the future.

## Conclusion

In the study, we confirmed that Compared with OSA-MCIs and HCs, OSA-nMCIs have higher dALFF values in the bilateral posterior cerebellar lobe and left superior frontal gyrus, which indicated that they have higher time variability and may be in an intermediate cognitive state, and an appropriate treatment of the disease may lead to different outcomes. The correlation between abnormal brain regions of OSA-MCI patients suggests that there is an abnormality in the cerebellar-prefrontal cortex circuit in patients with OSA-MCI; however, there are statistical correlations among the posterior cerebellar lobe and AHI, ESS score, microarousal index, and lowest SO2, suggesting that it may be used as an imaging marker of OSA. These findings deepen our understanding of the relationship between OSA and cognitive impairment.

## Data availability statement

The datasets presented in this study can be found in online repositories. The names of the repository/repositories and accession number(s) can be found in the article/Supplementary material.

## Ethics statement

The studies involving human participants were reviewed and approved by the Medical Ethics Committee of the First Affiliated Hospital of Nanchang University. The patients/participants provided their written informed consent to participate in this study.

## Author contributions

YS wrote, reviewed, and revised the manuscript. DP guided and designs the MRI experiment. LC analyzed the resting-state fMRI data. YS and LC analyzed and discussed the ideas of the manuscript. HL, LK, PL, WX, and YZ collected the resting fMRI data and applied for the ethics. All authors contributed to the article and approved the submitted version.

## Footnotes

1
www.mocatest.org

2
http://www.fil.ion.ucl.ac.uk

3
http://www.restfmri.net
