# Supplementary material for: Abnormal cerebellar-prefrontal cortical pathways in obstructive sleep apnea with/without mild cognitive impairment
Source: Front Neurosci. 2022 Oct 20;16:1002184. doi: 10.3389/fnins.2022.1002184 (PMC9630600; doi:10.3389/fnins.2022.1002184)
Supplement: Supplementary file 1 [file Image_1.pdf]

Considering that the initial correlation score showed the existence of 1-2 abnormal points, we tried to eliminate these two abnormal points and carried out a new correlation analysis after strictly checking the information of the subjects. In the OSA-nMCI group, the dALFF value of the left posterior cerebellum was significantly positively correlated with the AHI score (r = 0.565, p = 0.004), ESS score (r = 0.695, p <0.000), and arousal index (r = 0.460, p = 0.024), whereas the dALFF value of the right posterior cerebellar lobe was negatively correlated with the lowest oxygen saturation (r = -0.470, p = 0.021) (**Supplemental Material Figure 1**).

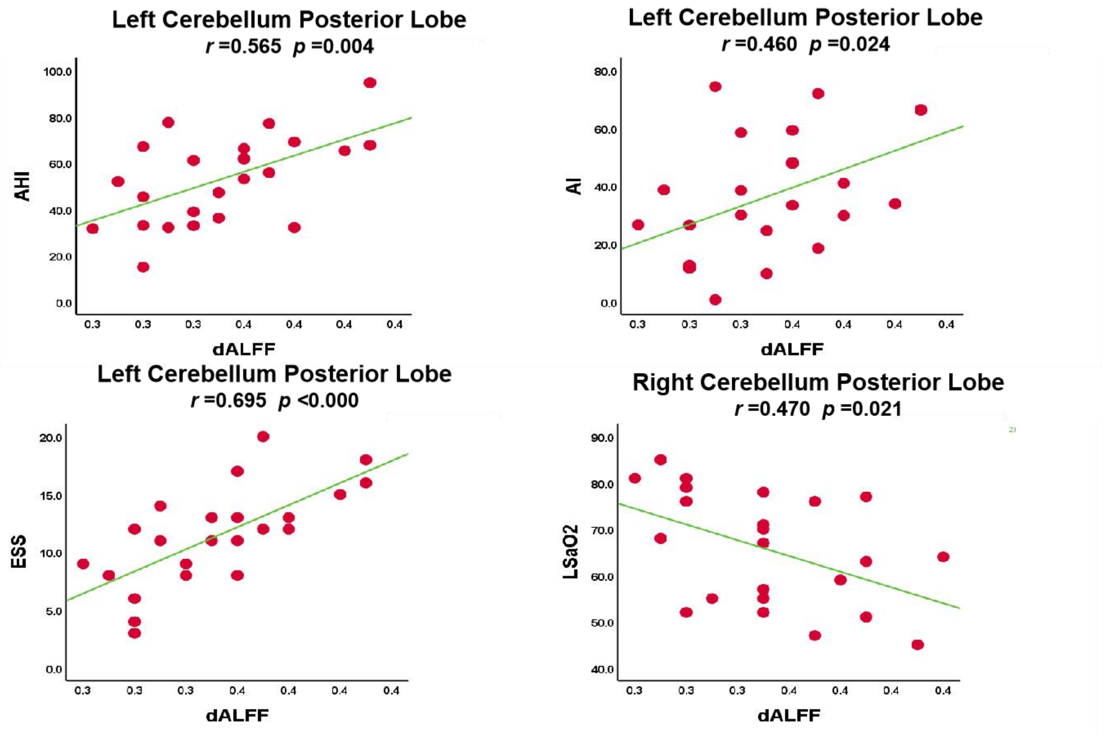

Supplemental Material Figure 1 Correlations between dALFF value and clinical information in OSA-nMCI after removing two outliers.
